# Deep learning-assisted lesion segmentation in PET/CT imaging: A feasibility study for salvage radiation therapy in prostate cancer

**DOI:** 10.18632/oncoscience.603

**Published:** 2024-05-20

**Authors:** Richard L.J. Qiu, Chih-Wei Chang, Xiaofeng Yang

**Affiliations:** ^1^Department of Radiation Oncology and Winship Cancer Institute, Emory University, Atlanta, GA 30322, USA

**Keywords:** PSMA PET/CT, deep learning, auto-segmentation, radiation oncology, prostate cancer

Prostate cancer persists as the most frequently diagnosed malignancy in men beyond skin cancer. Despite substantial advancements in treatment outcomes over the past half century, progression or recurrence post-initial treatments like prostatectomy or radiation therapy remains a challenge for a subset of patients. In those scenarios, salvage radiation therapy is often offered to patients as a treatment option. To design the salvage radiation therapy, imaging is required to detect and locate the recurrence disease regime. Traditional imaging modalities employed post-prostatectomy, such as CT, bone scans, MRI or ^18^F-FDG PET, often fall short in accurately detecting and determining the volume of the recurrent disease, which is crucial for salvage radiation treatment planning [1]. However, the introduction of ^18^F-fluciclovine (anti-1-amino-3-^18^F-fluorocyclobutane-1-carboxylic acid) PET/CT has marked a significant advancement in salvage disease management. Recent studies, including the phase 2/3 randomized controlled trial, Emory Molecular Prostate Imaging for Radiotherapy Enhancement (EMPIRE-1) [2], demonstrated improved biochemical recurrence or persistence free survival rates when incorporating ^18^F-fluciclovine PET/CT into post-prostatectomy radiation therapy planning.

One key step in salvage radiation therapy planning is the delineation of lesions on the ^18^F-fluciclovine PET/CT images, a task currently undertaken manually by physicians. This practice, while meticulous, is labor-intensive and prone to inter- and intra-observer variations. With the recent explosion of using artificial intelligence (AI) algorithms in medical image processing, automatic segmentation of lesions using deep learning (DL)-based lesion delineation methods [3] demonstrate promising potential to improve treatment quality, as appose to manual contouring. This editorial explores the research study by Wang et al. [4], showcasing the feasibility of DL models in lesion segmentation on ^18^F-fluciclovine PET/CT images.

The study utilized a cohort of 84 prostate cancer patients enrolled in Arm B of EMPIRE-1 trial, all of whom underwent ^18^F-fluciclovine PET/CT imaging. The authors proposed a novel cascaded detection segmentation network and benchmarked against two different neural networks—U-net and Cascaded U-net^3^—the research highlighted the capability of the proposed DL model to not only detect but also accurately delineate the lesions. The results revealed that their proposed DL model, which gave the best results, could achieve mean Dice similarity coefficient (DSC) of 0.68 ± 0.15, mean 95th percentile Hausdorff distance (HD95) of 4 ± 2 mm, mean center-of-mass distance (CMD) of 2.0 ± 1.5 mm, and mean volume difference (VD) of 0.6 ± 0.9 cc. It showed substantial agreement with manual contours drawn by expert radiologists and radiation oncologists.

The advantages of DL methods in lesion segmentation are multifold, mitigating the human contour variability and extensive time investment of manual contouring. Automated lesion segmentation acts as a vital corroborative step, enhancing the precision of treatment planning, reducing errors, and potentially improving patient outcomes through salvage radiation therapy. Nonetheless, the study’s findings also present several limitations that warrants further investigation. The observation that adding CT imaging did not enhance performance compared to PET alone is quite puzzling, as conventional wisdom suggests that more imaging adds information and typically yields better results. The study was limited to data from a single institution using the same PET/CT scanner, focusing primarily on small-volume lesions. Additionally, it did not consider the partial volume effects in PET imaging. Moreover, the study did not evaluate the clinical impact of automated segmentation on treatment outcomes, leaving a crucial aspect of clinical utility unaddressed.

The deployment of DL segmentation methods in ^18^F-fluciclovine PET/CT imaging represents an intriguing research direction for precision medicine in salvage prostate cancer care. With ongoing enhancements in these DL models, interdisciplinary collaboration among radiologists, radiation oncologists, medical physicists, computer scientists, and engineers becomes vital to maximize the potential of AI in cancer diagnosis and therapy planning. These efforts aim to refine diagnostic precision, optimize therapeutic strategies, and ultimately, elevate the standard of patient care. This research not only broadens our understanding of the technological capabilities but also reiterates the necessity for robust, multi-faceted approaches in handling complex clinical scenarios.

## References

[1] Odewole OA, et al. Eur J Nucl Med Mol Imaging. 2016; 43:1773–83. 10.1007/s00259-016-3383-8. 27091135 PMC4970909

[2] Jani AB, et al. Lancet. 2021; 397:1895–904. 10.1016/S0140-6736(21)00581-X. 33971152 PMC8279109

[3] Matkovic LA, et al. Phys Med Biol. 2021; 66:245006. 10.1088/1361-6560/ac3c13. 34808603 PMC8725511

[4] Wang T, et al. Front Oncol. 2023; 13:1274803. 10.3389/fonc.2023.1274803. 38156106 PMC10753832

